# Leelamine Modulates STAT5 Pathway Causing Both Autophagy and Apoptosis in Chronic Myelogenous Leukemia Cells

**DOI:** 10.3390/biology11030366

**Published:** 2022-02-25

**Authors:** Young Yun Jung, Jae-Young Um, Arunachalam Chinnathambi, Chandramohan Govindasamy, Gautam Sethi, Kwang Seok Ahn

**Affiliations:** 1 Department of Science in Korean Medicine, Kyung Hee University, Seoul 02447, Korea; ve449@naver.com (Y.Y.J.); jyum@khu.ac.kr (J.-Y.U.); 2 Department of Botany and Microbiology, College of Science, King Saud University, Riyadh 11451, Saudi Arabia; carunachalam@ksu.edu.sa; 3 Department of Community Health Sciences, College of Applied Medical Sciences, King Saud University, Riyadh 11433, Saudi Arabia; cgovindasamy@ksu.edu.sa; 4 Department of Pharmacology, Yong Loo Lin School of Medicine, National University of Singapore, Singapore 117600, Singapore

**Keywords:** leelamine, apoptosis, autophagy, STAT5, leukemia

## Abstract

**Simple Summary:**

Autophagy is a cellular mechanism that is essential for removing misfolded proteins and damaged organelles. Moreover, the aberrant activation of signal transducer and activator of transcription 5 (STAT5), which can regulate cellular survival and homeostasis, has been often observed in different malignancies. In this study, we demonstrate that leelamine inhibits the STAT5 phosphorylation while inducing autophagy as well as apoptosis in chronic myeloid leukemia cells. Leelamine induces autophagy by stimulating the expression of Atg7, beclin-1, and the production of autophagosomes, which leads to substantial inhibition of STAT5 activation.

**Abstract:**

Leelamine (LEE) has recently attracted significant attention for its growth inhibitory effects against melanoma, breast cancer, and prostate cancer cells; however, its impact on hematological malignancies remains unclear. Here, we first investigate the cytotoxic effects of LEE on several human chronic myeloid leukemia (CML) cells. We noted that LEE stimulated both apoptosis and autophagy in CML cells. In addition, the constitutive activation of signal transducer and activator of transcription 5 (STAT5) was suppressed substantially upon LEE treatment. Moreover, STAT5 knockdown with small interfering RNA (siRNA) increased LEE-induced apoptosis as well as autophagy and affected the levels of various oncogenic proteins. Thus, the targeted mitigation of STAT5 activation by LEE can contribute to its diverse anticancer effects by enhancing two distinct cell death pathways.

## 1. Introduction

Chronic myelogenous leukemia (CML) is characterized by the presence of the Philadelphia chromosome (Ph), which arises due to the reciprocal translocation of the (9;22) chromosome and can lead to the formation of bcr-abl [1]. Bcr-abl is an oncogenic kinase that has been found to induce the activation of MEK/ERK, PI3K, and JAK/STAT signaling pathways and promote aberrant proliferation and survival [2,3,4,5,6]. When CML enters the myeloid blast crisis phase from the chronic phase, additional mutations have been found in the genes that are related to bcr-abl that can induce the disease progression. Several tyrosine kinase inhibitors, such as imatinib, that can target Bcr-abl, have been successfully applied for disease control in CML patients. However, although TKIs were found to be effective against the chronic phase of the disease, some patients have reported relapse as a result of the development of drug resistance [7]. These findings have indicated the necessity to develop novel strategies to effectively treat CML patients.

Autophagy is an intracellular degradative process that can occur as a result of poor nutritional and hypoxic conditions or chemotherapy treatment [8,9,10,11]. Autophagy is a vital cellular process for removing misfolded proteins and various organelles that might be resistant to apoptosis [12,13]. During the autophagy process, autophagosomes can mediate the intracellular degradation of various proteins or aggregates. The formation of autophagosomes is vital for the recycling of degraded cytoplasmic components by the fusion with lysosomes during stressful conditions. The activation of autophagy has also been found to be essential for preservation of the cellular metabolism and survival in different cancers [14,15,16].

Signal transducer and activator of transcription 5 (STAT5) can exhibit important functions to regulate cellular survival and homeostasis; however, it can be often aberrantly activated in different tumor cells [17,18,19]. STAT5 is one of the STAT family members that has been closely related to the development of various malignancies [20,21,22,23]. In fact, constitutive phosphorylation of STAT5 in diverse cancers has been reported in several previous studies [24,25,26,27]. The phosphorylation of STAT5 can be stimulated through the phosphorylation of distinct kinases such as JAK1, JAK2, and Src [28,29]. Additionally, in our previous study, we reported that suppression of STAT5 phosphorylation can promote apoptosis activation in lung cancer cells [30]. Moreover, another previous study has suggested that STAT5 can inhibit the autophagy pathway in mesangial cells [31].

Leelamine (LEE) is one of the identified lysosomotropic compounds obtained from the bark of pine trees [32,33,34]. Interestingly, the inhibitory effects of LEE on pyruvate dehydrogenase kinases have been already reported and its suppressive actions on the proliferation of melanoma, breast cancer, and prostate cancer cells have been recently identified, but its influence on CML cells remain unclear [32,33,35,36,37,38,39,40].

It has also been found previously that several existing pharmacological inhibitions of autophagy or Atg 5 and 7 knockdown can induce the activation of apoptosis in CML cells [41]. Apoptosis is another necessary process for maintaining and protecting the various intracellular components [42,43,44]. Both autophagy and apoptosis play a critical role in protecting against cellular damage and chronic conditions, including cancer. Natural products have been found to eliminate cancer cells by promoting both apoptosis and autophagy [45,46]. Thus, in this study, we aimed to investigate whether LEE can effectively induce autophagy as well as apoptosis and the potential interaction between these two cell death pathways. We found that LEE can stimulate both autophagy and apoptosis almost simultaneously, and a crosstalk was noted between these two processes. Additionally, LEE suppressed STAT5 activation through inhibiting upstream signals such as JAK1/2 and Src activation. Our study suggests that LEE can display significant anticancer potential against CML through diverse molecular mechanisms.

## 2. Materials and Methods

### 2.1. Reagents

**Figure 1:**
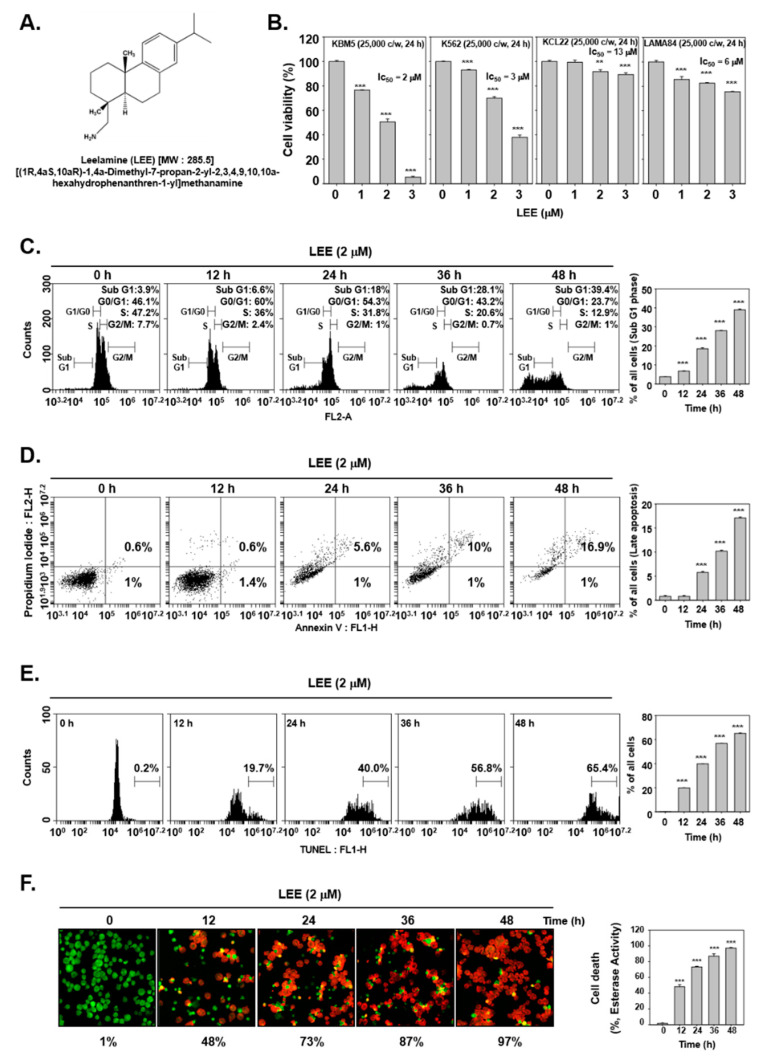
Effects of LEE on apoptosis induction in KBM5 cells. (**A**) Chemical structure of LEE (Leelamine). (**B**) KBM5, K562, KCL22, and LAMA84 cells (2.5 × 10^4^ cells/well) were treated with LEE for 24 h, then cell viability was measured by MTT assay. (**C**) KBM5 cells (5 × 10^5^ cells/well) were treated with LEE (2 μm) for indicated time intervals (0, 12, 24, 36, 48 h). Cells were digested with RNase A for 1 h, and stained with propidium iodide (PI) and then analyzed by flow cytometric analysis. (**D**) LEE (2 μm) treated KBM5 cells were incubated with Annexin V-FITC and PI for 15 min. Apoptosis was measured by flow cytometric analysis. (**E**) KBM5 cells were treated with LEE, then fixed and stained with TUNEL assay reagent. (**F**) Live and dead assay was performed with KBM5 cells. *** *p* < 0.001 vs. non-treated (NT) cells, ** *p* < 0.01 vs. non-treated (NT) cells vs. non-treated (NT) cells.

Leelamine (LEE, Figure 1A) was purchased from Cayman Chemical (Ann Arbor, MI, USA). LEE stock solution (10 mm) was prepared in dimethyl sulfoxide, storage at −20 °C and finally diluted in cell culture medium to use. Fetal bovine serum (FBS) and penicillin-streptomycin mixture were purchased from Thermo Fisher Scientific Inc. (Waltham, MA, USA). TUNEL (terminal transferase-mediated dUTP-fluorescein nick end labeling) assay kit was from Roche Diagnostics GmbH (Mannheim, Germany).

### 2.2. Cell Lines and Culture Conditions 

KBM5, K562, KCL22, and LAMA84 cells were obtained from American Type Culture Collection (Manassas, VA, USA). KBM5 cells were grown in IMDM medium containing 10% FBS with 1% penicillin/streptomycin. K562, KCL22, and LAMA84 cells were cultured in RPMI 1640 medium containing 10% FBS with 1% penicillin/streptomycin. 

### 2.3. MTT Assay

KBM5, K562, KCL22, and LAMA84 cells (2.5 × 10^4^ cells/well) were exposed to LEE (0, 1, 2, 3 μm) for 24 h. To measure cell viability MTT assay was conducted as described previously [47]. The half-maximal inhibitory concentration (IC50) was determined based on cell viability.

### 2.4. Western Blot Analysis

After KBM5 cells were treated with indicated various concentrations of the drug, cells were harvested and whole cell lysates were obtained. Thereafter, Western blot was performed as described in our earlier study [48].

### 2.5. RT-PCR Analysis 

To confirm the mRNA expression levels of different genes, RNA was extracted from LEE-treated KBM5 cells. Extracted RNA was reverse transcribed into cDNA, and then reverse transcription polymerase chain reaction (RT-PCR) was performed as described previously [49].

### 2.6. Live/Dead Assay

Apoptosis was also examined by live/dead assay as indicated earlier [50]. KBM5 cells exposed to LEE were stained with 5 μm of calcein-AM and Ethd-1(Ethidium homodimer-1) at 37 °C for 30 min.

### 2.7. Acridine Orange Assay 

To evaluate the autophagy activation, acridine staining was performed as described previously [50].

### 2.8. MDC Staining

Monodansylcadaverine (MDC) staining was also performed to measure increasing of acid vesicular organelles by LEE in KBM5 cells. Indicated time and concentrations treated cells were stained with MDC (50 μm) for 20 min at 37 °C. Then cells were attached to the slide glass by cytospin. Cells were detected by Olympus FluoView FV1000 confocal microscope (Tokyo, Japan).

### 2.9. TUNEL Assay

To evaluate LEE-induced cell death, TUNEL assay was performed as described earlier [50].

### 2.10. Immunocytochemistry for LC3 Expression 

To evaluate LEE-induced LC3 expression, immunocytochemistry was performed as described previously [51].

### 2.11. Knockdown of Beclin-1 and Atg7 Expression by siRNA Transfection 

KBM5 cells were transfected with 50 nm of Beclin-1, Atg7, and scrambled siRNAs for 48 h in penicillin/streptomycin-free IMDM media by using Neon™ Transfection System (Invitrogen, Carlsbad, CA, USA). After transfection, cells were treated with LEE (2 μm) for 48 h in complete media and analysed for various assays. 

### 2.12. Blockage of STAT5 Expression by siRNA 

Briefly, KBM5 cells transfected with STAT5 and scrambled siRNA (50 nm) with Neon™ Transfection System (Invitrogen, Carlsbad, CA, USA). Scrambled siRNA was used as positive control.

### 2.13. Bcl-2 Overexpression by pEGFP Bcl-2 Transfection 

To over express Bcl-2 levels, the cells were transfected with pEGFP-Bcl-2 plasmid in penicillin/streptomycin-free IMDM media using by Neon™ Transfection System (Invitrogen, Carlsbad, CA, USA) for 24 h. Then the cells were treated with LEE (2 μm) for 48 h in complete media and whole cell extracts were prepared for Western blot. 

### 2.14. Statistical Analysis

All the numerical values have been represented as the mean ± SD. Statistical significance of the data compared with the untreated control was determined using the Student’s unpaired *t*-test. Significance was set at * *p*  <  0.05, ** *p*  <  0.01, and *** *p*  <  0.001. All experiments were performed independently at least 3 times and representative data are shown.

## 3. Results

### 3.1. LEE-Promoted Cell Death through Causing Apoptosis 

First, we confirmed the effects of LEE on the viability of the various leukemic cells (KBM5, K562, KCL22, and LAMA84) by MTT assay. We found that LEE significantly reduced the cell viability in K562 and LAMA84 cells but only slightly in KCL22 cells. Especially in KBM5 cells, cell viability showed a significant decrease upon LEE treatment. Thus, we selected the KBM5 cells as the representative cells for further experiments (Figure 1B). Moreover, a 2 μm dose of LEE was selected with reference to its IC50 value. Thereafter, we analyzed the impact of LEE on cell death using a live/dead assay. Figure 1F shows that as LEE treatment time increased, the number of red cells also increased. This observation suggests that LEE-treated cells displayed cell death in a time-dependent fashion.

Based on cell viability decrease, we examined whether LEE-induced cell death results in apoptosis. KBM5 cells were exposed to LEE and the cell cycle progression was analyzed. The results suggested that the percentage of cells stagnated in sub-G1 increased proportionately with increasing concentrations of the drug (Figure 1C). Next, we studied apoptosis by the annexin V assay. PI and annexin V-FITC stained cells were sorted by flow cytometer into live, necrotic, early apoptosis, or late apoptosis populations. The LEE-treated cells showed increased concentration in the late apoptosis stage (Figure 1D). Thereafter, we detected the terminal transferase (TdT) probed 3′-OH termini of the DNA fragment by a TUNEL assay. The number of TdT-labelled cells was also found to be markedly increased. (Figure 1E).

### 3.2. LEE-Triggered Autophagy via Production of Acidic Vesicular Organelles 

**Figure 2:**
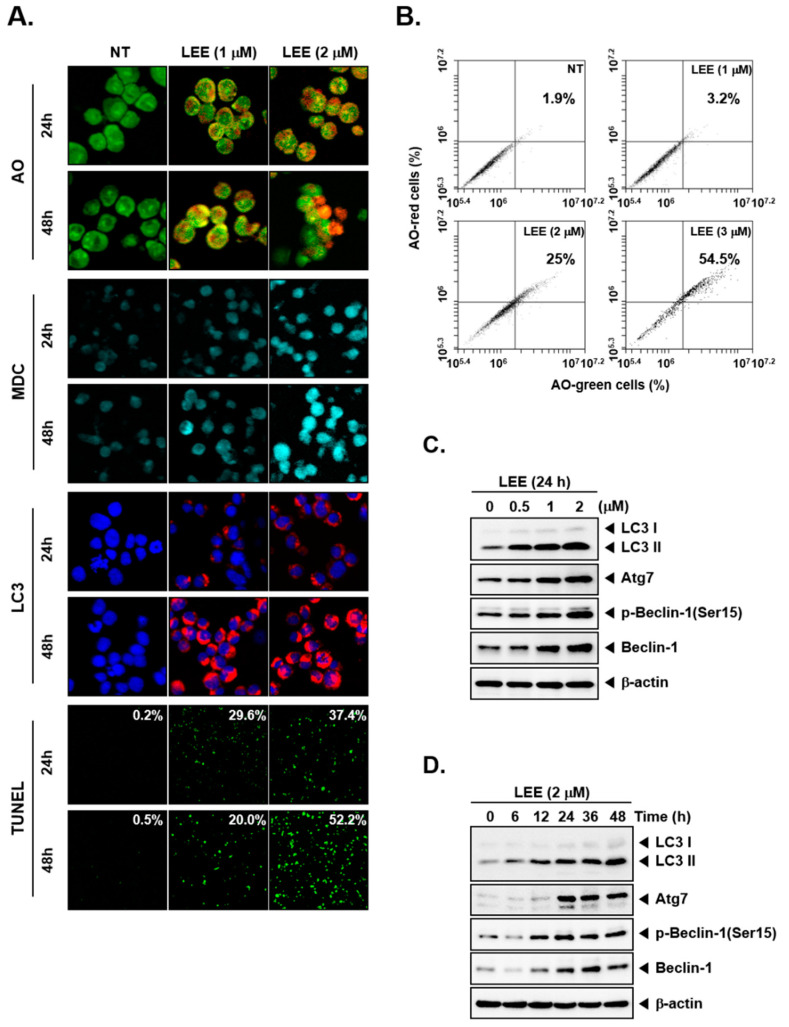
Induction of autophagy by LEE. (**A**) KBM5 cells (5 × 10^5^ cells/well) were treated with LEE (0, 1, and 2 μm) for 24 or 48 h. Autophagy was detected by AO, MDC staining or probed with LC3 antibody for immunocytochemistry. (**B**) Autophagy was measured by AO staining. KBM5 cells were treated with LEE (0, 1, 2, and 3 μm) for 48 h and analyzed by cell flow cytometry. (**C**) KBM5 cells were treated with LEE (0, 0.5, 1, and 2 μm) for 24 h and autophagy markers were measured by Western blot analysis. (**D**) KBM5 cells were treated with LEE (2 μm) for indicated time intervals (0, 6, 12, 24, 36, and 48 h) and proteins were evaluated by Western blot analysis.

To confirm the production of acidic vesicular organelles, which are considered a representative feature of autophagy, we performed MDC or acridine orange (AO) staining, which can selectively stain acidic components. When autophagy was induced, it was found that acidic components were stained in orange, so we established the activation of autophagy by an increase in AO-stained cells. Moreover, MDC stained autophagic vacuoles within the cytoplasm and nucleus and caused them to appear as the light blue dots (Figure 2A). We also analyzed AO-stained cells by flow cytometer. It was found that the percentage of AO-stained cells increased with increased concentration (Figure 2B). According to these findings, it was established that LEE caused autophagy with acidic vesicular organelle production. 

Because LC3II is important for autophagosome formation and maturation, we examined whether LEE could induce the LC3 expression by immunocytochemistry. After the LEE treatment, KBM5 cells were probed with an anti-LC3 antibody. As shown in Figure 2A, the expression of LC3 was increased by LEE treatment and a 2 μm dose yielded the highest expression along with increased TUNEL staining. Then we analyzed whether LEE could increase the levels of autophagy related markers. It was observed that expression levels of LC3, Atg7, and Beclin 1 were substantially increased upon exposure to LEE (Figure 2C,D and Figure S1).

### 3.3. Modulation of Various Cell Survival Proteins by LEE 

**Figure 3:**
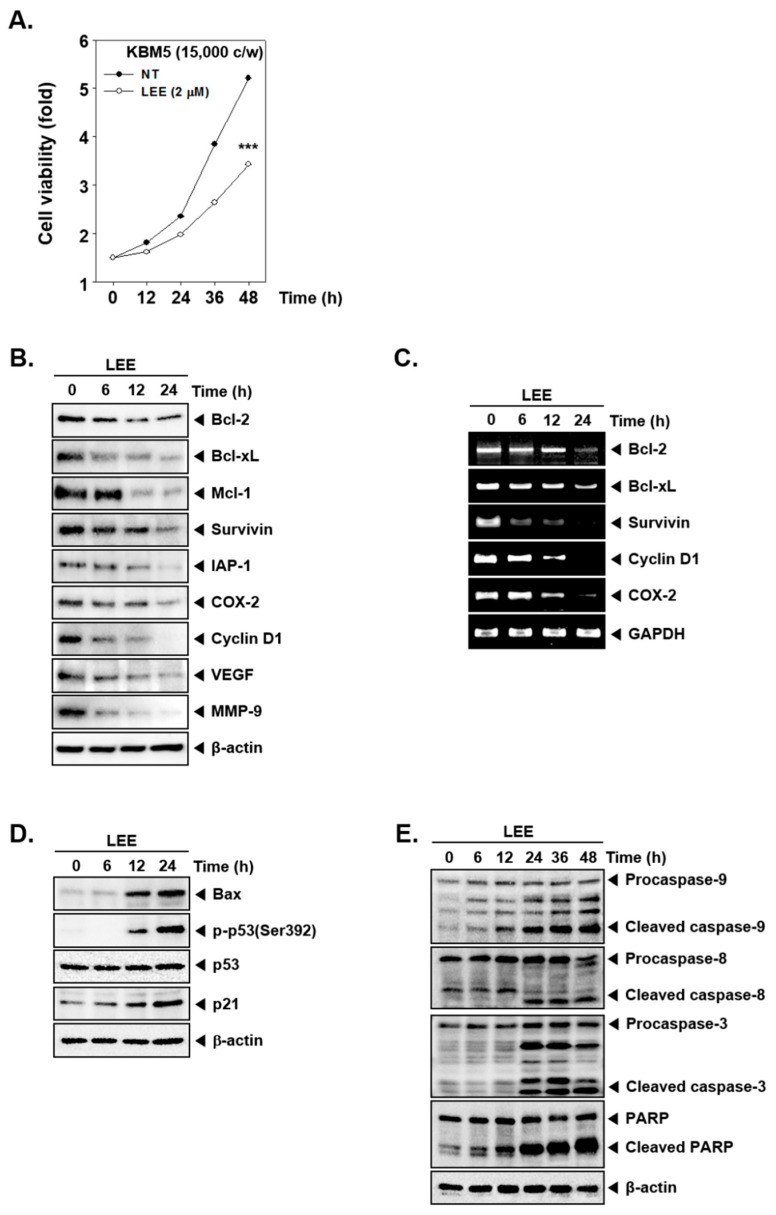
Analysis of levels of various proteins upon LEE treatment. (**A**) The effect on cell proliferation in KBM5 cells upon exposure to LEE. KBM5 cells (1.5 × 10^4^ cells/well) were treated by 2 μm of LEE for 12 h time intervals and MTT assay was performed. *** *p* < 0.001. (**B**) KBM5 cells were treated by LEE (2 μm) for 0, 6, 12, and 24 h, and levels of various proteins were evaluated by Western blot analysis. (**C**) mRNA levels of selected genes were examined by RT-PCR. (**D**) LEE (2 μm) treated KBM5 cells were evaluated for expression of various proteins by Western blot analysis. (**E**) KBM5 cells were treated with LEE (2 μm), then apoptosis markers were evaluated by Western blot analysis.

We next measured the impact of LEE on cell viability by the MTT assay. As shown in Figure 3A, compared with the non-treated cells, LEE suppressed the cell viability significantly. Then we investigated the levels of various oncogenic proteins by Western blot analysis. It was noted that LEE substantially reduced expression levels of Bcl-2, Bcl-xl, Mcl-1, survivin, IAP-1, COX-2, cyclin D1, VEGF, and MMP-9 proteins (Figure 3B and Figure S2). Additionally, we also selected some representative markers and determined the effect of the drug on their mRNA levels. It was noted that mRNA expression of all these markers displayed a decreasing pattern upon LEE treatment (Figure 3C).

We also examined the levels of Bax, p53, and p21 in LEE-treated cells by Western blot analysis. Bax has been related to apoptosis induction and p21 can cause cell cycle arrest, whereas p53 can induce cell death as well as autophagy. As shown in Figure 3D (Figure S3), LEE augmented the Bax, p-p53, and p21 expressions in a time-dependent fashion. The results suggested that LEE can induce substantial cell death.

To confirm the apoptosis, we evaluated the caspase-9, 8, 3, and PARP cleavage by Western blot analysis. Caspase-9, 8, 3, and PARP were found to be activated, as evidenced by the formation of cleaved products. It was noted that expression of these proteins was clearly triggered after 24 h of treatment (Figure 3E and Figure S3).

### 3.4. LEE-Induced Cell Death through Autophagy and Apoptosis Even in the Presence of Different Pharmacological Inhibitors

**Figure 4:**
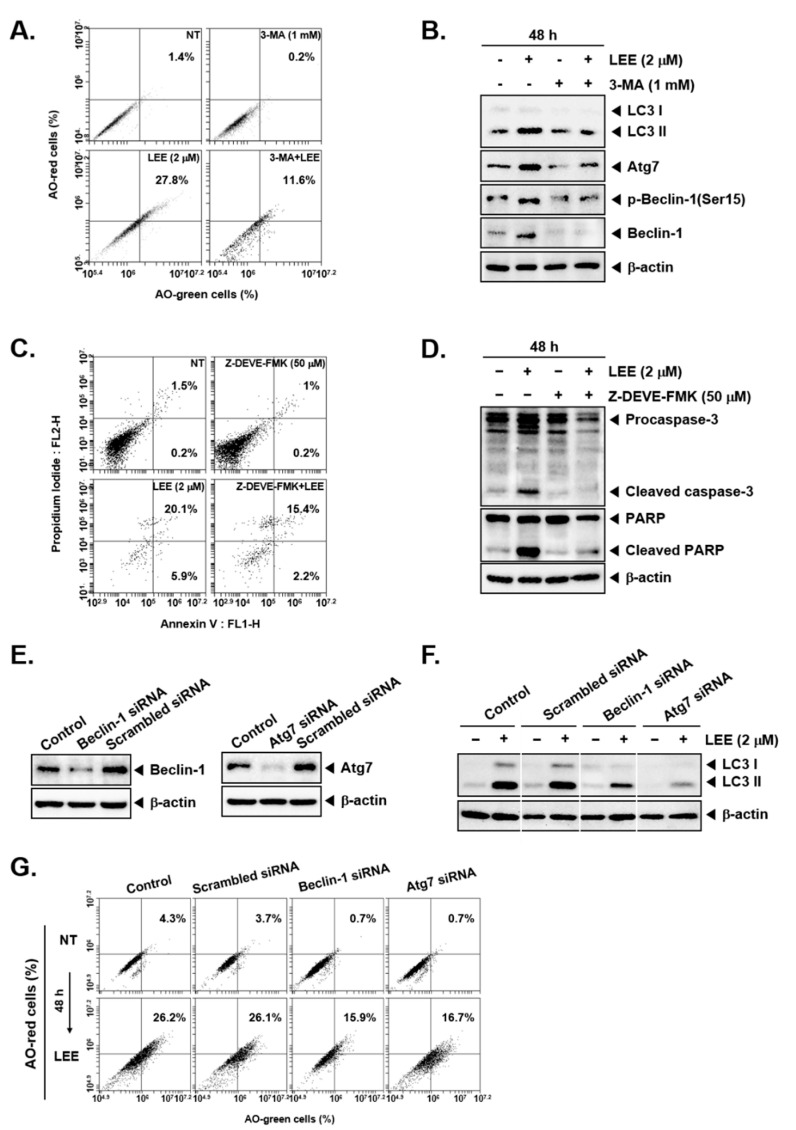
Effect of LEE in combination with pharmacological blockers. (**A**) KBM5 cells (5 × 10^5^ cells/well) were treated with autophagy inhibitor, 1 mm of 3-Methyladenine (3-MA) and 2 μm of LEE for 48 h. Cells were stained with acridine orange and analyzed by cell flow cytometry. (**B**) 3-MA and LEE treated KBM5 cells were evaluated for expression of various autophagy related proteins. (**C**) KBM5 cells (5 × 10^5^ cells/well) were treated with caspase-3 inhibitor, 50 μm of Z-DEVE-FMK and 2 μm of LEE for 48 h. The cells were labelled with annexin-FITC for 15 min, and then analyzed by cell flow cytometry. (**D**) Z-DEVE-FMK and LEE treated KBM5 cells were examined for caspase-3 and PARP expression by Western blot. (**E**) Beclin-1 and Atg7 proteins were knocked down by siRNA transfection. (**F**) The expression of Beclin-1 or Atg7 proteins in KBM5 cells was knocked out by siRNA transfection. The cells were then treated with LEE (2 μm) for 48 h and LC3 levels were analyzed by Western blot. (**G**) The cells were processed as described in F and then subjected to AO assay.

To confirm whether LEE-induced cell death is a result of autophagy activation, we treated KBM5 cells with 1 mm of 3-Methyladenine (3-MA), an autophagy inhibitor, and LEE for 48 h. The results indicated that LEE significantly induced autophagy, but 3-MA and LEE co-treated cells displayed reduced effectiveness in causing autophagy as compared to the treatment with LEE alone (Figure 4A). Thereafter, we evaluated whether the levels of autophagy-related proteins were affected by 3-MA. It was found that expression of LC3II, Atg7, p-Beclin, and Beclin was suppressed by 3-MA and also expression levels of these proteins could be recovered to some extent upon co-treatment with LEE (Figure 4B and Figure S4). These results demonstrate that LEE induced autophagy through modulating the levels of LC3, atg-7, p-beclin-1, and beclin-1.

We next confirmed whether apoptosis induced by LEE treatment was mediated primarily by a caspase-3 dependent pathway. Hence, we treated 50 μm of Z-DEVE-FMK, a caspase-3 inhibitor, and LEE for 48 h. As shown in Figure 4C, Z-DEVE-FMK suppressed the effects of LEE, from 20.1% to 15.4%, on apoptosis activation. It suggested that the effect of LEE on apoptosis was inhibited by Z-DEVE-FMK, thus suggesting that it induced cell death via caspase activation. Then we also confirmed whether caspase-3 and PARP cleavages were affected by Z-DEVE-FMK by using Western blot analysis. LEE significantly increased the cleavage of both caspase-3 and PARP proteins. In addition, attenuation caused by Z-DEVE-FMK showed some recovery upon co-treatment with LEE (Figure 4D and Figure S4).

Since beclin-1, Atg7, and LC3 activity in autophagy have been found to be linked, we confirmed the effect of knocking down beclin-1 and Atg7 on LC3 levels. We knocked down expression of Beclin-1 and Atg7 by siRNA, and then treated the cells with LEE for 48 h. When the cells were transfected with Beclin-1 and Atg7 by siRNA, expression of LC3II was suppressed and the effects of LEE were also reduced (Figure 4E,F and Figure S5). Thereafter, we analyzed the cells by AO staining and the results suggested that the knockdown of beclin-1 and Atg7 clearly decreased autophagy activation (Figure 4G).

### 3.5. LEE-Induced Autophagy and Apoptosis via Driving STAT5 Inhibition

**Figure 5:**
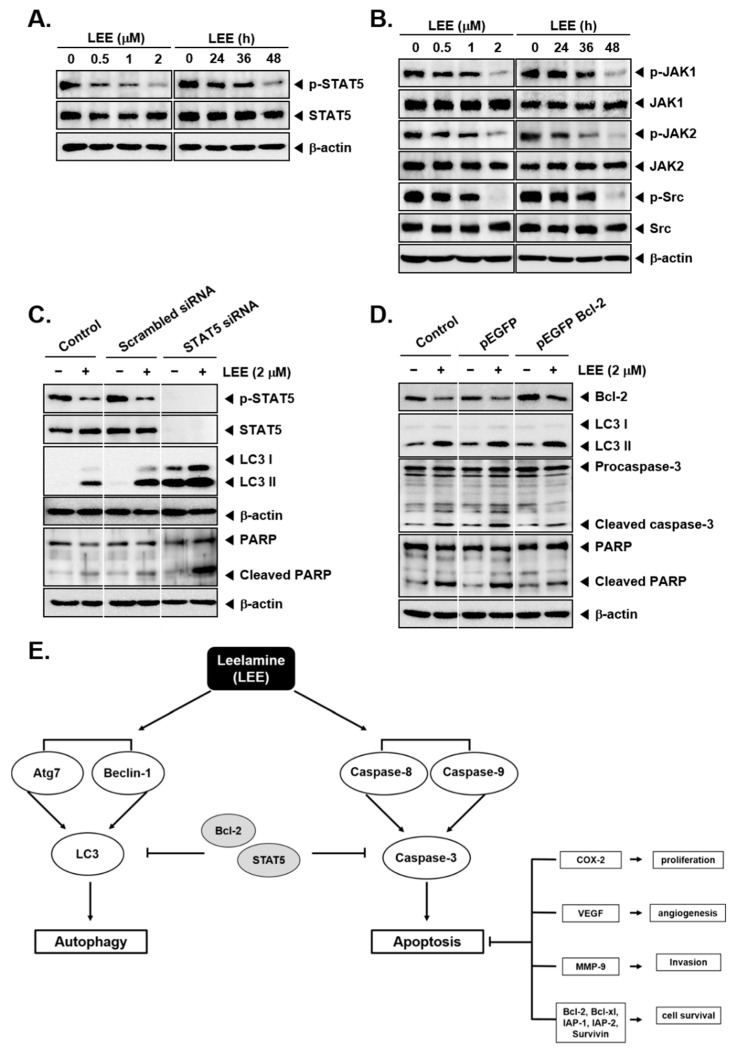
Inhibition of STAT5 activation by LEE. (**A**,**B**) KBM5 cells were treated by LEE with indicated concentration and time-dependent conditions. Phosphorylation of STAT5 and upstream signals such as JAK1, JAK2, and Src was evaluated by Western blot. (**C**) KBM5 cells were transfected with STAT5 siRNA, and then treated with LEE (2 μm) for 48 h. p-STAT5, LC3, and PARP expression levels were evaluated by Western blot. (**D**) Analysis of Bcl-2, LC3, caspase-3, and PARP expression levels in LEE-treated Bcl-2 overexpressed cells by Western blot analysis. (**E**) A schematic diagram showing mode of action of LEE.

STAT5 activation plays an important role in CML progression, hence we investigated the effects of LEE on this pathway. As shown in Figure 5A (Figure S6), it was found that the phosphorylation of STAT5 was significantly decreased by LEE. Because JAK1, JAK2, and Src have been reported to act as upstream signals of STAT5 activity, we confirmed the impact of LEE on these kinases. The cells were treated with various concentration and time conditions and the results indicated that both concentrations and time conditions had a pronounced inhibition of various kinases (Figure 5B and Figure S7).

We investigated whether STAT5 suppression can affect autophagy and apoptosis induced by LEE by causing STAT5 gene silencing with siRNA. STAT5 siRNA transfection knocked down the expression of STAT5 protein but increased the levels of LC3II and PARP, which potentially indicated augmented effects of LEE on the expression of both autophagy and apoptosis-related markers (Figure 5C and Figure S8).

### 3.6. Overexpression of bcl-2 Reduced Autophagy and Apoptosis

Thereafter, we confirmed the impact of LEE on various markers upon overexpression of Bcl-2 after transfection. It was observed that the levels of the apoptosis makers such as cleaved caspase-3 and PARP expression were suppressed, though there was no effect on LC3II levels (Figure 5D and Figure S9).

## 4. Discussion

Our goal in this study was to investigate the modulation of apoptosis, autophagy, and the STAT5 pathway by LEE in CML cells. A few studies have reported the potential interaction between autophagy, apoptosis, and STAT5 in different cancer cell lines [30,31,41]. Because resistance to apoptosis has been known to play an important role in tumor development ls [51,52], we examined the effect of LEE on apoptosis activation [53,54]. Natural products have been demonstrated to target various cancer hallmarks in different tumor models [55,56]. A number of important proteins, such as caspase-9, 8, 3, and PARP, have been implicated in the process of apoptotic cell death [57]. We conducted a cell cycle analysis as well as TUNEL and annexin V assays to study apoptosis. The concentration of LEE was based on the IC50 value, and our results showed that the cell cycle progression was arrested in the G1-phase and an increased number of apoptotic cells were observed as the treatment time was increased. Moreover, LEE induced PARP and caspase cleavage while suppressing the expression of anti-apoptotic proteins. The findings suggest that LEE can significantly suppress tumorigenesis through stimulating apoptosis in CML cells [58].

Autophagy is another important strategy to induce cell death in organisms [12,13,59]. LC3 is a protein that can effectively conjugate with LC3I and LC3II to form the autophagosome, so LC3II can be potentially used as an autophagosomal marker [60,61,62]. ATG7 is known as an important autophagy-related protein along with ATG5, which can play a key role in causing activation of autophagy [63,64,65]. In our study, autophagy was induced in a dose and time-dependent fashion. We found that LEE activated autophagosomes through AO as well as MDC staining. LC3 I, LC3 II, and Atg7 expression were also induced by LEE. Interestingly, deletion of Atg7 and Beclin 1 expression by siRNA transfection reduced the increased levels of both LC3 I and LC3 II, thereby providing sound evidence about the autophagy induction potential of LEE. Moreover, both apoptosis and autophagy have been reported to play an important role in maintaining homeostasis by diverse mechanisms [66,67,68]. Autophagy can protect cells from apoptotic cell death by inhibiting apoptosis [69]. On the contrary, suppression of autophagy can lead to an increase in apoptosis [70,71]. However, in our study, LEE induced apoptosis and autophagy at the same time and can exhibit its anti-neoplastic actions through modulating the levels of various apoptosis and autophagy related proteins.

Uncontrolled STAT5 activation plays a pivotal role in regulating cancer cell proliferation and invasion, so targeted inhibition of STAT5 can be a useful approach for cancer therapy [17,18,19]. Our findings indicated that LEE significantly suppressed the phosphorylation of STAT5 and up-stream kinases in CML cells. Interestingly, the deletion of STAT5 by using siRNA substantially enhanced the effect of LEE on the expressions of both apoptosis and autophagy-related proteins. These findings indicate that LEE not only affected STAT5 phosphorylation but also STAT5-induced apoptosis and autophagy activation.

In conclusion, this study demonstrated that LEE can effectively induce CML cell death through stimulating both apoptosis and autophagy pathways. Moreover, LEE also attenuated STAT5 activation to inhibit the growth and survival of CML cells. Therefore, LEE can be further developed as an effective therapeutic for the management of CML and other malignancies.

## Data Availability

All data is freely available with this article.

## Supplementary Materials

The following supporting information can be downloaded at: https://www.mdpi.com/article/10.3390/biology11030366/s1, Figure S1. Western blot membrane of LC3, Atg7, p-beclin-1, beclin-1, and b-actin proteins were detected with anti-LC3 (12741s; 1:3000; Cell signaling), anti-Atg7 (85585s; 1:3000; Cell signaling), anti-p-beclin-1 (84966s; 1:3000; Cell signaling), anti-beclin-1 (4122s; 1:3000; Cell signaling), and anti-b-actin (sc47778; 1:5000; Santacruz) antibodies. Proteins were separated on different percentages of gels, and transferred to nitrocellulose membranes (66485; Pall Corporation). Then Membranes were incubated with mouse anti-goat IgG HRP (sc2354; 1:5000; Santacruz), mouse anti-rabbit IgG HRP (sc2357; 1:5000; Santacruz), mouse-IgGkHRP (sc516102; 1:5000; Santacruz) secondary antibody on room-temperature. Finally membranes were developed with LumiFemtosolution (DG-WF200; 1:1; DoGeNBio). Figure S2. Western blot membrane of Bcl-2, Bcl-xl, Mcl-1, survivin, IAP-1, Cox-2, Cyclin D1, VEGF, MMP-9 and b-actin proteins were detected with anti-Bcl-2(sc-492; 1:3000; Santacruz), anti-Bcl-xl(sc-7195: 1;3000; Santacruz), anti-Mcl-1(sc-819; 1:3000; Santacruz), anti-survivin(sc-17779; 1:3000; Santacruz), anti-IAP-1 (sc-7943; 1:3000; Santacruz), anti-Cox-2 (sc-19999; 1:3000; Santacruz), anti-Cyclin D1 (2978s; 1:3000; Cell signaling), anti-VEGF (sc-7269; 1:3000; Santacruz), anti-MMP-9 (sc-393859; 1:3000; Santacruz), and anti-b-actin (sc-47778; 1:5000; Santacruz) antibodies. Proteins were separated on different percentages of gels, and transferred to nitrocellulose membranes (66485; Pall Corporation). Then Membranes were incubated with mouse anti-goat IgG HRP (sc2354; 1:5000; Santacruz), mouse anti-rabbit IgG HRP (sc2357; 1:5000; Santacruz), mouse-IgGkHRP (sc516102; 1:5000; Santacruz) secondary antibody on room-temperature. Finally membranes were developed with LumiFemtosolution (DG-WF200; 1:1; DoGeNBio). Figure S3. Western blot membrane of (A) Bax, p-p53, p53, p21, and b-actin, (B) caspase-9, caspase-8, caspase-3, PARP and b-actin proteins were detected with anti-Bax(sc-7480; 1:3000; Santacruz), anti-p-p53 (sc-7997; 1:3000; Santacruz), anti-p53 (sc-6243; 1:3000; Santacruz), anti-p21 (sc-817; 1:3000; Santacruz), anti-caspase-9 (9505s; 1:3000; Cell signaling), anti-caspase-8 (9496s; 1:3000; Cell signaling), anti-caspase-3 (9661s; 1:3000; Cell signaling), anti-PARP (sc-8007; 1:3000; Santacruz)and anti-b-actin (sc-47778; 1:5000; Santacruz) antibodies. Proteins were separated on different percentages of gels, and transferred to nitrocellulose membranes (66485; Pall Corporation). Then Membranes were incubated with mouse anti-goat IgG HRP (sc2354; 1:5000; Santacruz), mouse anti-rabbit IgG HRP (sc2357; 1:5000; Santacruz), mouse-IgGkHRP (sc516102; 1:5000; Santacruz) secondary antibody on room-temperature. Finally membranes were developed with LumiFemtosolution (DG-WF200; 1:1; DoGeNBio). Figure S4. Western blot membrane of (A) LC3, Atg7, p-beclin-1, beclin-1, and b-actin, (B) caspase-3, PARP and b-actin proteins were detected with anti-LC3 (12741s; 1:3000; Cell signaling), anti-Atg7 (85585s; 1:3000; Cell signaling), anti-p-beclin-1 (84966s; 1:3000; Cell signaling), anti-beclin-1 (4122s; 1:3000; Cell signaling), anti-caspase-3 (9661s; 1:3000; Cell signaling), anti-PARP (sc-8007; 1:3000; Santacruz) and anti-b-actin (sc-47778; 1:5000; Santacruz) antibodies. Proteins were separated on different percentages of gels, and transferred to nitrocellulose membranes (66485; Pall Corporation). Then Membranes were incubated with mouse anti-rabbit IgG HRP (sc2357; 1:5000; Santacruz), mouse-IgGkHRP (sc516102; 1:5000; Santacruz) secondary antibody on room-temperature. Finally membranes were developed with LumiFemtosolution (DG-WF200; 1:1; DoGeNBio). Figure S5. Western blot membrane of Atg7, beclin-1, LC3, and b-actin proteins were detected with anti-Atg7 (85585s; 1:3000; Cell signaling), anti-beclin-1 (4122s; 1:3000; Cell signaling), anti-LC3 (12741s; 1:3000; Cell signaling), and anti-b-actin (sc-47778; 1:5000; Santacruz) antibodies. Proteins were separated on different percentages of gels, and transferred to nitrocellulose membranes (66485; Pall Corporation). Then Membranes were incubated with mouse anti-rabbit IgG HRP (sc2357; 1:5000; Santacruz), mouse-IgGkHRP (sc516102; 1:5000; Santacruz) secondary antibody on room-temperature. Finally membranes were developed with LumiFemtosolution (DG-WF200; 1:1; DoGeNBio). Figure S6. Western blot membrane of p-STAT5, STAT5 and b-actin proteins were detected with anti-p-STAT5 (9314s; 1:3000; Cell signaling), anti-STAT5 (sc-74442; 1:3000; Santacruz) and anti-b-actin (sc-47778; 1:5000; Santacruz) antibodies. Proteins were separated on different percentages of gels, and transferred to nitrocellulose membranes (66485; Pall Corporation). Then Membranes were incubated with mouse anti-rabbit IgG HRP (sc2357; 1:5000; Santacruz), mouse-IgGkHRP (sc516102; 1:5000; Santacruz) secondary antibody on room-temperature. Finally membranes were developed with LumiFemtosolution (DG-WF200; 1:1; DoGeNBio). Figure S7. Western blot membrane of p-JAK1, JAK1, p-JAK2, JAK2, p-Src, Src, and b-actin proteins were detected with anti-p-JAK1 (3331s; 1:2000; Cell signaling), anti-JAK1 (3332s; 1:2000; Cell signaling), anti-p-JAK2 (3776s; 1:2000; Cell signaling), anti-JAK2 (3230s; 1:2000; Cell signaling), anti-p-src(2101s; 1:2000; Cell signaling), anti-src(sc-5266; 1:3000; Santacrus) and anti-b-actin (sc-47778; 1:5000; Santacruz) antibodies. Proteins were separated on different percentages of gels, and transferred to nitrocellulose membranes (66485; Pall Corporation). Then Membranes were incubated with mouse anti-rabbit IgG HRP (sc2357; 1:5000; Santacruz), mouse-IgGkHRP (sc516102; 1:5000; Santacruz) secondary antibody on room-temperature. Finally membranes were developed with LumiFemtosolution (DG-WF200; 1:1; DoGeNBio). Figure S8. Western blot membrane of p-STAT5, STAT5, LC3, PARP, and b-actin proteins were detected with anti-p-STAT5 (9314s; 1:3000; Cell signaling), anti-STAT5 (sc-74442; 1:3000; Santacruz), anti-LC3 (12741s; 1:3000; Cell signaling), anti-PARP (sc-8007; 1:3000; Santacruz), and anti-b-actin (sc-47778; 1:5000; Santacruz) antibodies. Proteins were separated on different percentages of gels, and transferred to nitrocellulose membranes (66485; Pall Corporation). Then Membranes were incubated with mouse anti-rabbit IgG HRP (sc2357; 1:5000; Santacruz), mouse-IgGkHRP (sc516102; 1:5000; Santacruz) secondary antibody on room-temperature. Finally membranes were developed with LumiFemtosolution (DG-WF200; 1:1; DoGeNBio). Figure S9. Western blot membrane of Bcl-2, LC3, Caspase-3, PARP, and b-actin proteins were detected with anti-Bcl-2 (sc-492; 1:3000; Santacruz), anti-LC3 (12741s; 1:3000; Cell signaling),anti-Caspase-3 (9661s; 1:3000; Cell signaling), anti-PARP (sc-8007; 1:3000; Santacruz), and anti-b-actin (sc-47778; 1:5000; Santacruz). Proteins were separated on different percentages of gels, and transferred to nitrocellulose membranes (66485; Pall Corporation). Then Membranes were incubated with mouse anti-rabbit IgG HRP (sc2357; 1:5000; Santacruz), mouse-IgGkHRP (sc516102; 1:5000; Santacruz) secondary antibody on room-temperature. Finally membranes were developed with LumiFemtosolution (DG-WF200; 1:1; DoGeNBio).
