# Supplementary material for: Leelamine Modulates STAT5 Pathway Causing Both Autophagy and Apoptosis in Chronic Myelogenous Leukemia Cells
Source: Biology (Basel). 2022 Feb 25;11(3):366. doi: 10.3390/biology11030366 (PMC8945775; doi:10.3390/biology11030366)
Supplement: Supplementary file 1 [file biology-11-00366-s001.zip › biology-1576807-supplementary.pdf]

Supplementary Materials

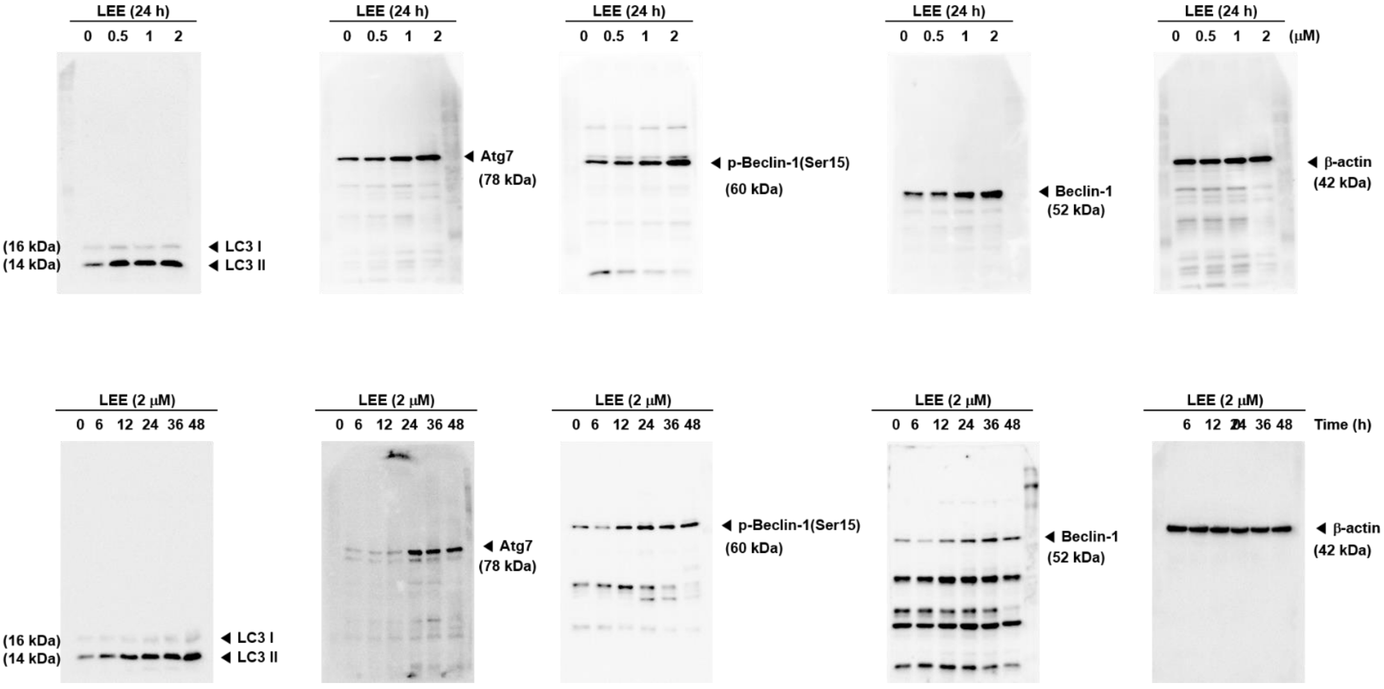

Figure S1. Western blot membrane of LC3, Atg7, p-beclin-1, beclin-1, and β-actin proteins were detected with anti- LC3 (12741s; 1:3000; Cell signaling), anti-Atg7 (85585s; 1:3000; Cell signaling), anti-p-beclin-1 (84966s; 1:3000; Cell signaling), anti-beclin-1 (4122s; 1:3000; Cell signaling), and anti-β-actin (sc47778; 1:5000; Santacruz) antibodies. Proteins were separated on different percentages of gels, and transferred to nitrocellulose membranes (66485; Pall Corporation). Then Membranes were incubated with mouse anti-goat IgG HRP (sc2354; 1:5000; Santacruz), mouse anti-rabbit IgG HRP (sc2357; 1:5000; Santacruz), mouse-IgGk HRP (sc516102; 1:5000; Santacruz) secondary antibody on room-temperature. Finally membranes were developed with Lumi Femto solution (DG-WF200; 1:1; DoGeNBio)

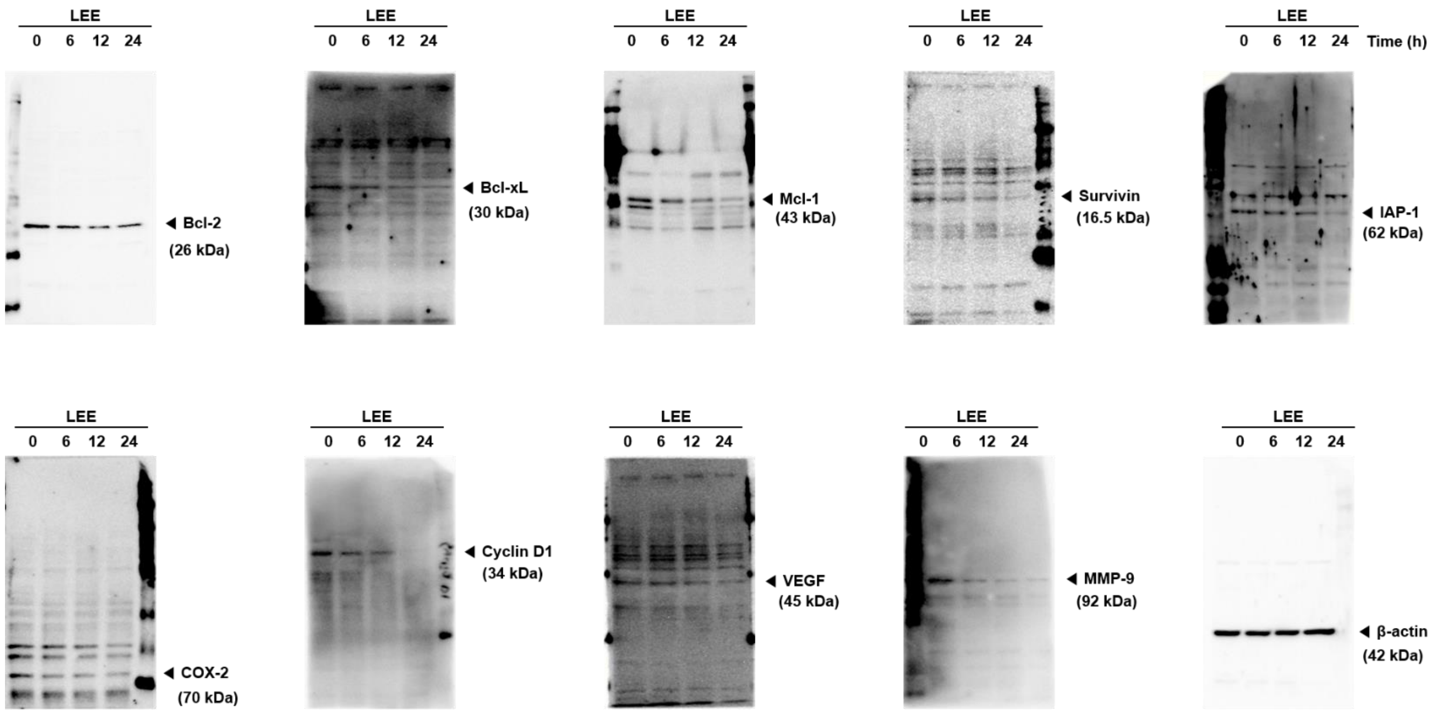

Figure S2. Western blot membrane of Bcl-2, Bcl-xl, Mcl-1, survivin, IAP-1, Cox-2, Cyclin D1, VEGF, MMP-9 and β-actin proteins were detected with anti- Bcl-2 (sc-492; 1:3000; Santacruz), anti- Bcl-xl (sc-7195: 1;3000; Santacruz), anti- Mcl-1 (sc-819; 1:3000; Santacruz), anti- survivin (sc-17779; 1:3000; Santacruz), anti-IAP-1 (sc-7943; 1:3000; Santacruz), anti-Cox-2 (sc-19999; 1:3000; Santacruz), anti-Cyclin D1 (2978s; 1:3000; Cell signaling), anti-VEGF (sc-7269; 1:3000; Santacruz), anti-MMP-9 (sc-393859; 1:3000; Santacruz), and anti-β-actin (sc-47778; 1:5000; Santacruz) antibodies. Proteins were separated on different percentages of gels, and transferred to nitrocellulose membranes (66485; Pall Corporation). Then Membranes were incubated with mouse anti-goat IgG HRP (sc2354; 1:5000; Santacruz), mouse anti-rabbit IgG HRP (sc2357; 1:5000; Santacruz), mouse-IgGk HRP (sc516102; 1:5000; Santacruz) secondary antibody on room-temperature. Finally membranes were developed with Lumi Femto solution (DG-WF200; 1:1; DoGeNBio)

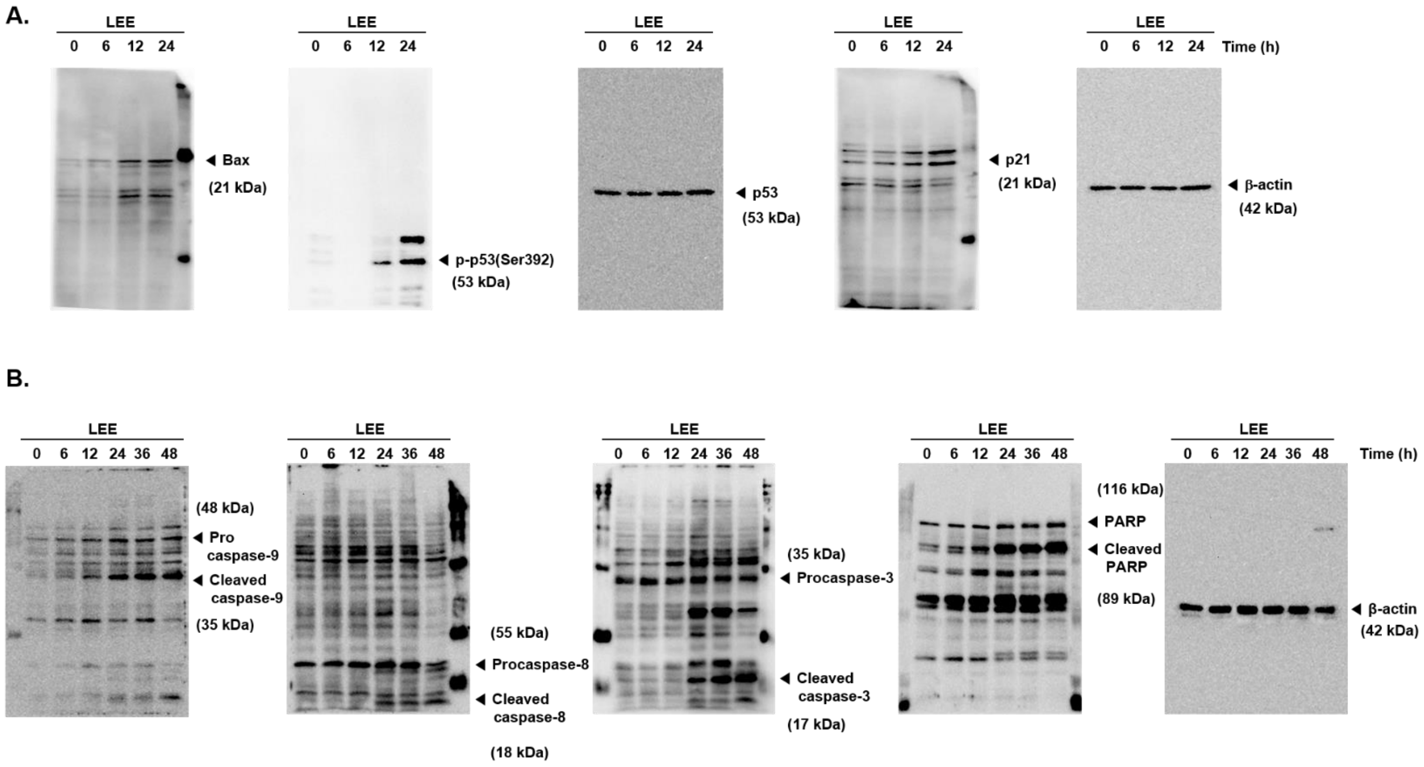

Figure S3. Western blot membrane of (A) Bax, p-p53, p53, p21, and β-actin, (B) caspase-9, caspase-8, caspase-3, PARP and β-actin proteins were detected with anti-Bax (sc-7480; 1:3000; Santacruz), anti-p-p53 (sc-7997; 1:3000; Santacruz), anti-p53 (sc-6243; 1:3000; Santacruz), anti-p21 (sc-817; 1:3000; Santacruz), anti-caspase-9 (9505s; 1:3000; Cell signaling), anti-caspase-8 (9496s; 1:3000; Cell signaling), anti-caspase-3 (9661s; 1:3000; Cell signaling), anti-PARP (sc-8007; 1:3000; Santacruz) and anti-β-actin (sc-47778; 1:5000; Santacruz) antibodies. Proteins were separated on different percentages of gels, and transferred to nitrocellulose membranes (66485; Pall Corporation). Then Membranes were incubated with mouse anti-goat IgG HRP (sc2354; 1:5000; Santacruz), mouse anti-rabbit IgG HRP (sc2357; 1:5000; Santacruz), mouse-IgGk HRP (sc516102; 1:5000; Santacruz) secondary antibody on room-temperature. Finally membranes were developed with Lumi Femto solution (DG-WF200; 1:1; DoGeNBio)

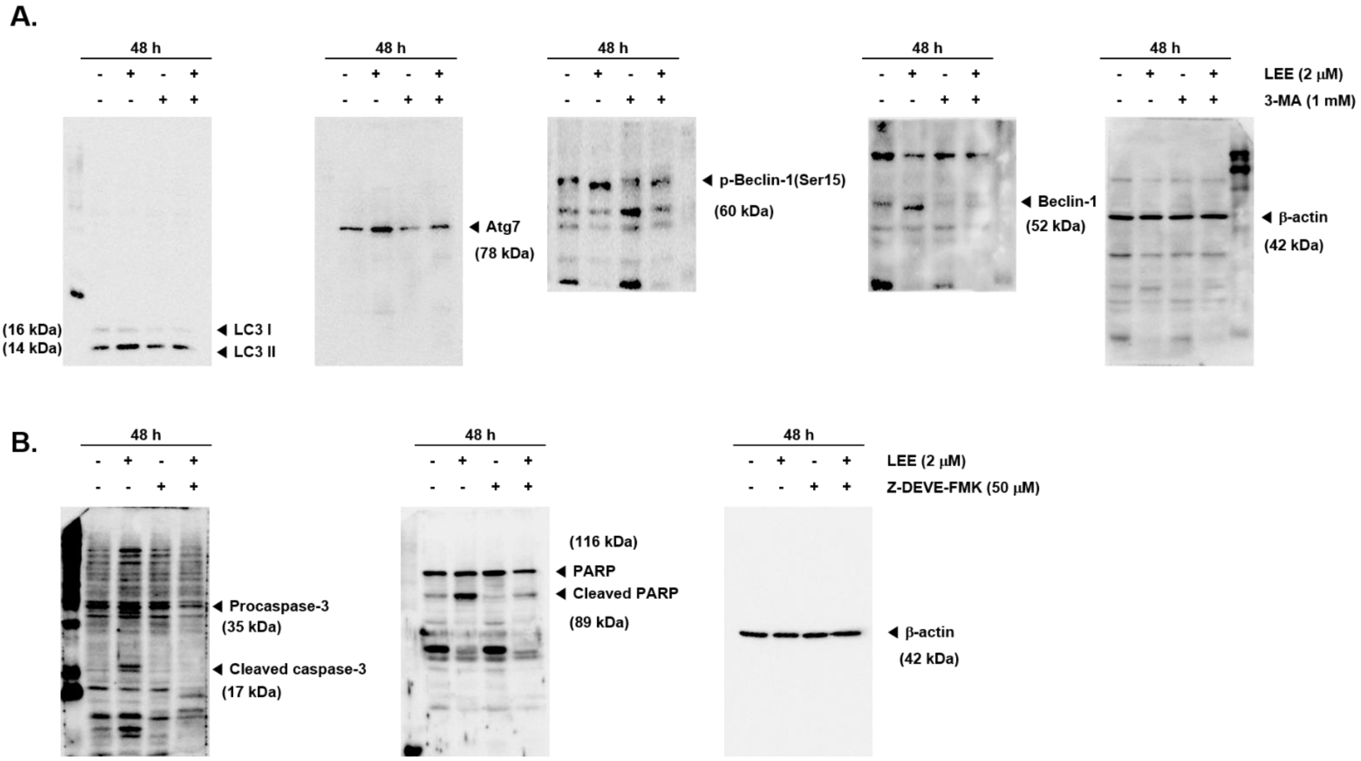

Figure S4. Western blot membrane of (A) LC3, Atg7, p-beclin-1, beclin-1, and β-actin, (B) caspase-3, PARP and β-actin proteins were detected with anti- LC3 (12741s; 1:3000; Cell signaling), anti-Atg7 (85585s; 1:3000; Cell signaling), anti-p-beclin-1 (84966s; 1:3000; Cell signaling), anti-beclin-1 (4122s; 1:3000; Cell signaling), anti-caspase-3 (9661s; 1:3000; Cell signaling), anti-PARP (sc-8007; 1:3000; Santacruz) and anti-β-actin (sc-47778; 1:5000; Santacruz) antibodies. Proteins were separated on different percentages of gels, and transferred to nitrocellulose membranes (66485; Pall Corporation). Then Membranes were incubated with mouse anti-rabbit IgG HRP (sc2357; 1:5000; Santacruz), mouse-IgGk HRP (sc516102; 1:5000; Santacruz) secondary antibody on room-temperature. Finally membranes were developed with Lumi Femto solution (DG-WF200; 1:1; DoGeNBio)

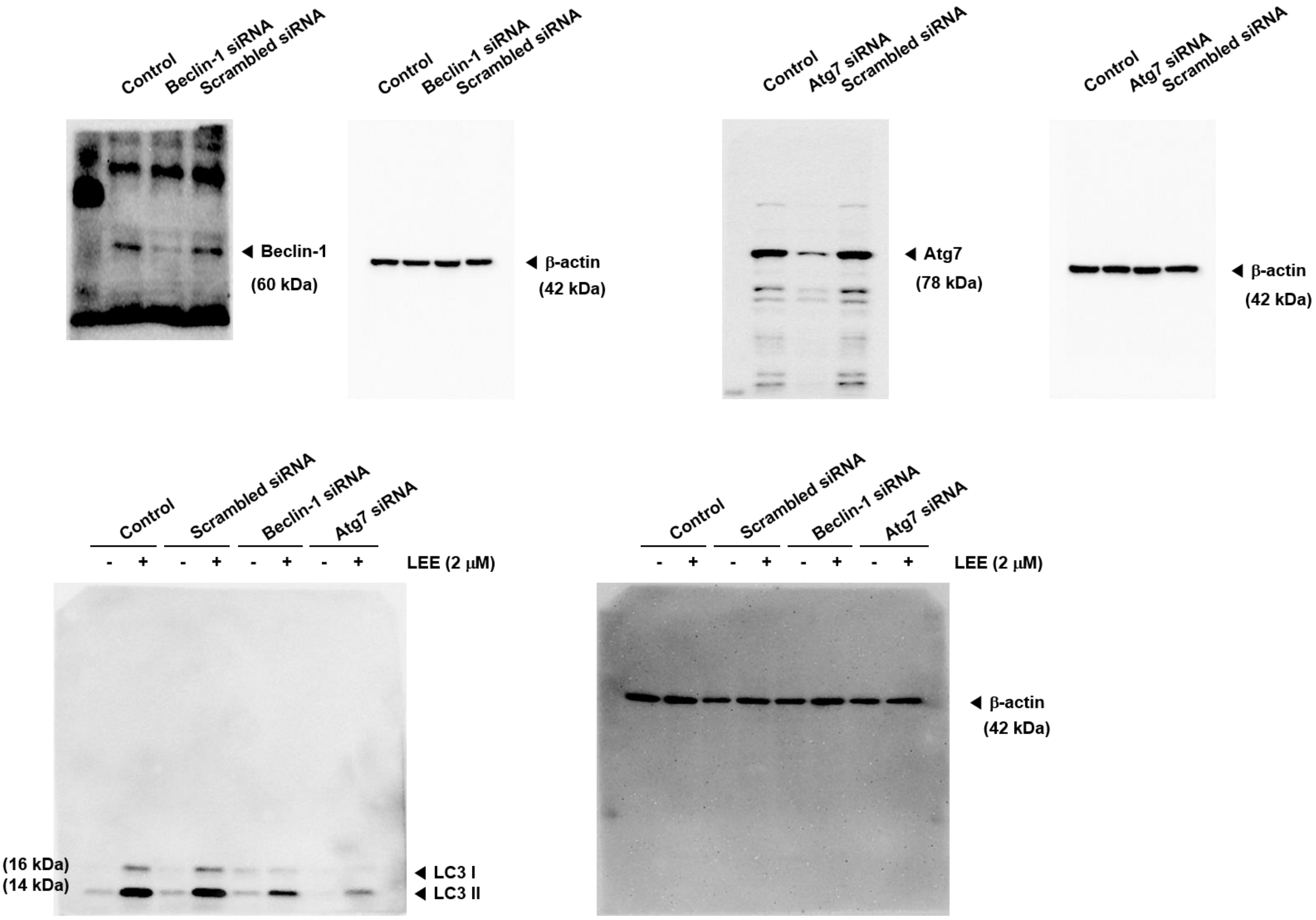

Figure S5. Western blot membrane of Atg7, beclin-1, LC3, and β-actin proteins were detected with anti-Atg7 (85585s; 1:3000; Cell signaling), anti-beclin-1 (4122s; 1:3000; Cell signaling), anti-LC3 (12741s; 1:3000; Cell signaling), and anti-β-actin (sc-47778; 1:5000; Santacruz) antibodies. Proteins were separated on different percentages of gels, and transferred to nitrocellulose membranes (66485; Pall Corporation). Then Membranes were incubated with mouse anti-rabbit IgG HRP (sc2357; 1:5000; Santacruz), mouse-IgGk HRP (sc516102; 1:5000; Santacruz) secondary antibody on room-temperature. Finally membranes were developed with Lumi Femto solution (DG-WF200; 1:1; DoGeNBio)

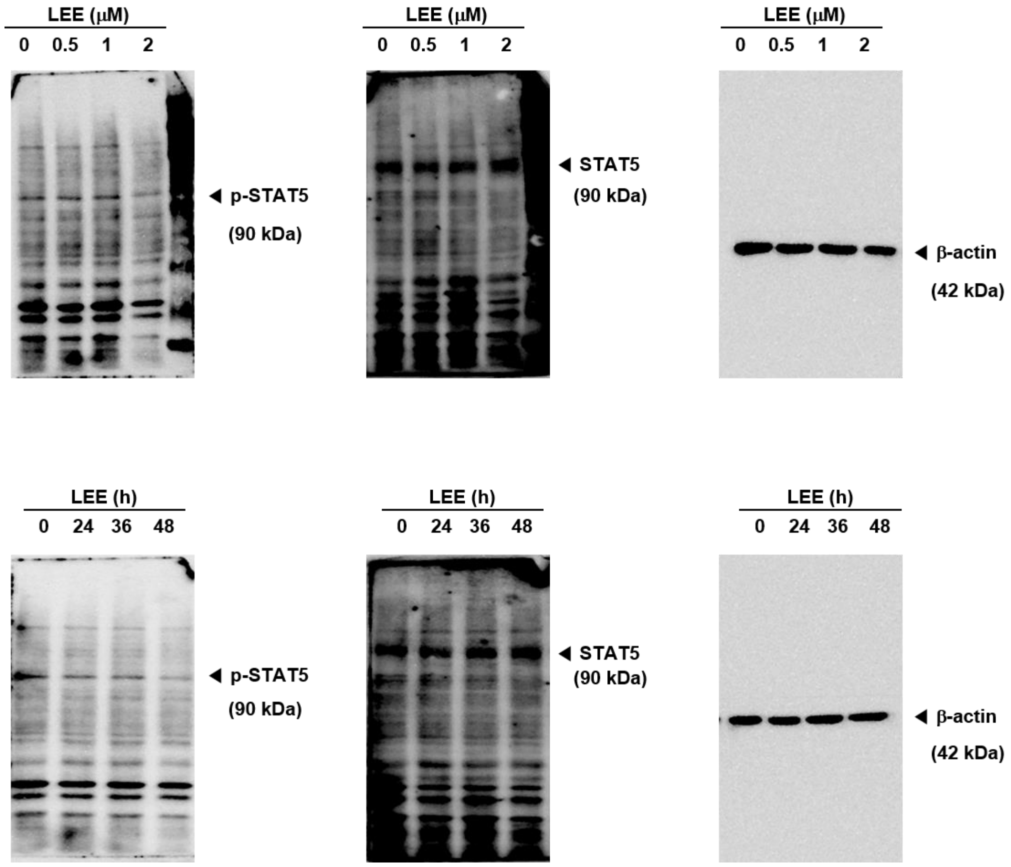

Figure S6. Western blot membrane of p-STAT5, STAT5 and β-actin proteins were detected with anti-p-STAT5 (9314s; 1:3000; Cell signaling), anti-STAT5 (sc-74442; 1:3000; Santacruz) and anti-β-actin (sc-47778; 1:5000; Santacruz) antibodies. Proteins were separated on different percentages of gels, and transferred to nitrocellulose membranes (66485; Pall Corporation). Then Membranes were incubated with mouse anti-rabbit IgG HRP (sc2357; 1:5000; Santacruz), mouse-IgGk HRP (sc516102; 1:5000; Santacruz) secondary antibody on room-temperature. Finally membranes were developed with Lumi Femto solution (DG-WF200; 1:1; DoGeNBio)

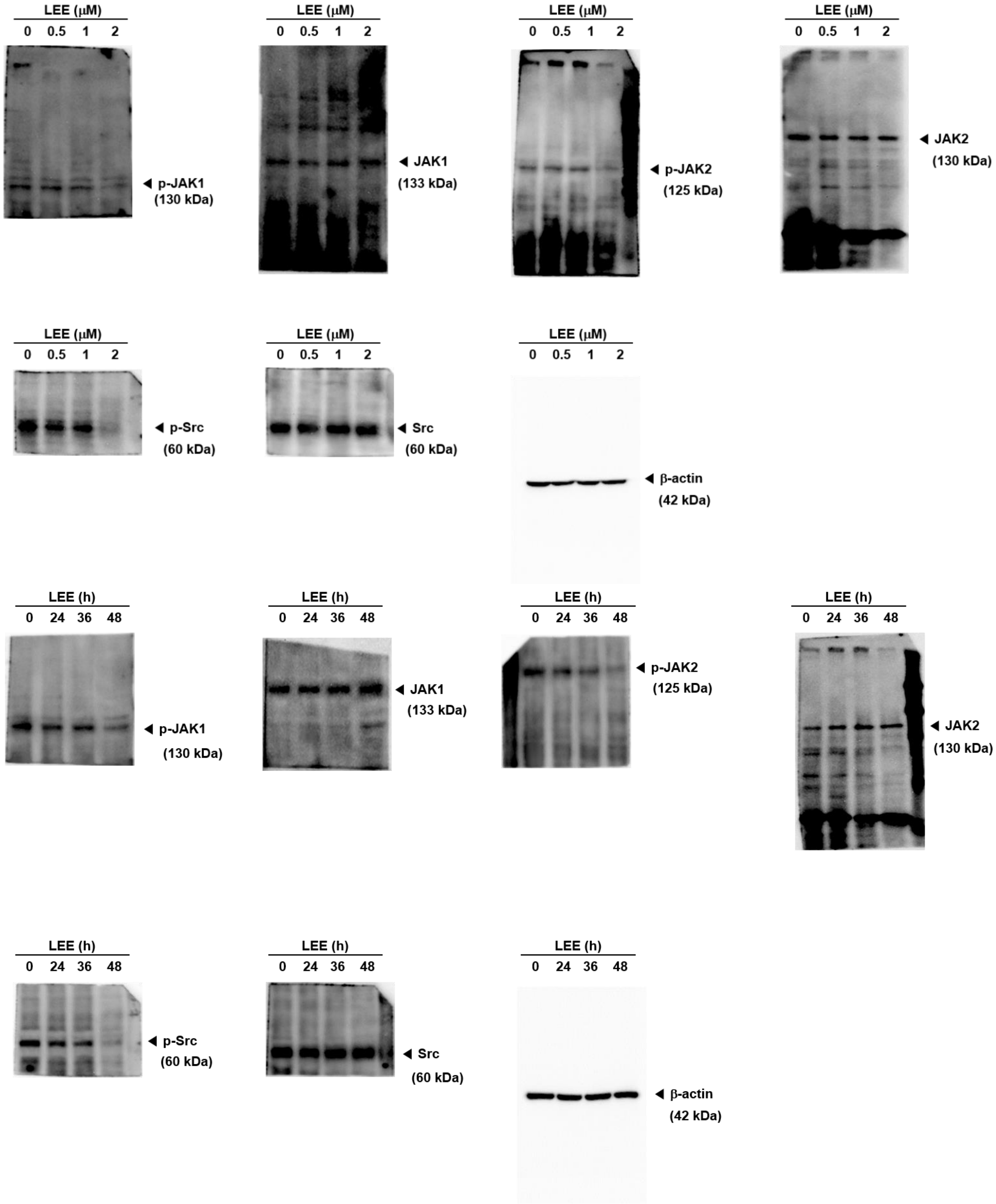

Figure S7. Western blot membrane of p-JAK1, JAK1, p-JAK2, JAK2, p-Src, Src, and β-actin proteins were detected with anti-p-JAK1 (3331s; 1:2000; Cell signaling), anti-JAK1 (3332s; 1:2000; Cell signaling), anti-p-JAK2 (3776s; 1:2000; Cell signaling), anti-JAK2 (3230s; 1:2000; Cell signaling), anti-p-src (2101s; 1:2000; Cell signaling), anti-src (sc-5266; 1:3000; Santacrus) and anti-β-actin (sc-47778; 1:5000; Santacruz) antibodies. Proteins were separated on different percentages of gels, and transferred to nitrocellulose membranes (66485; Pall Corporation). Then Membranes were incubated with mouse anti-rabbit IgG HRP (sc2357; 1:5000; Santacruz), mouse-IgGk HRP (sc516102; 1:5000; Santacruz) secondary antibody on room-temperature. Finally membranes were developed with Lumi Femto solution (DG-WF200; 1:1; DoGeNBio)

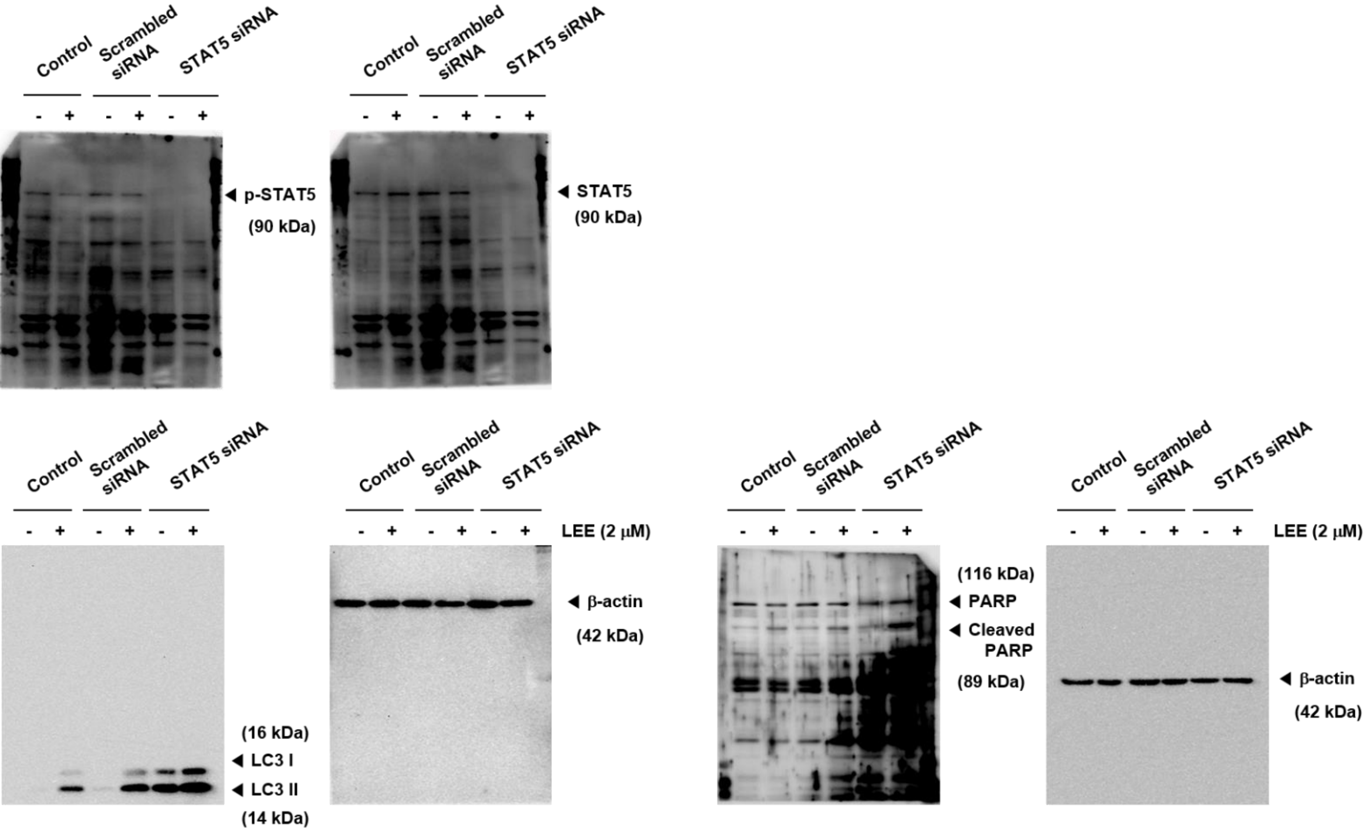

Figure S8. Western blot membrane of p-STAT5, STAT5, LC3, PARP, and β-actin proteins were detected with anti-p-STAT5 (9314s; 1:3000; Cell signaling), anti-STAT5 (sc-74442; 1:3000; Santacruz), anti- LC3 (12741s; 1:3000; Cell signaling), anti-PARP (sc-8007; 1:3000; Santacruz), and anti-β-actin (sc-47778; 1:5000; Santacruz) antibodies. Proteins were separated on different percentages of gels, and transferred to nitrocellulose membranes (66485; Pall Corporation). Then Membranes were incubated with mouse anti-rabbit IgG HRP (sc2357; 1:5000; Santacruz), mouse-IgGk HRP (sc516102; 1:5000; Santacruz) secondary antibody on room-temperature. Finally membranes were developed with Lumi Femto solution (DG-WF200; 1:1; DoGeNBio)

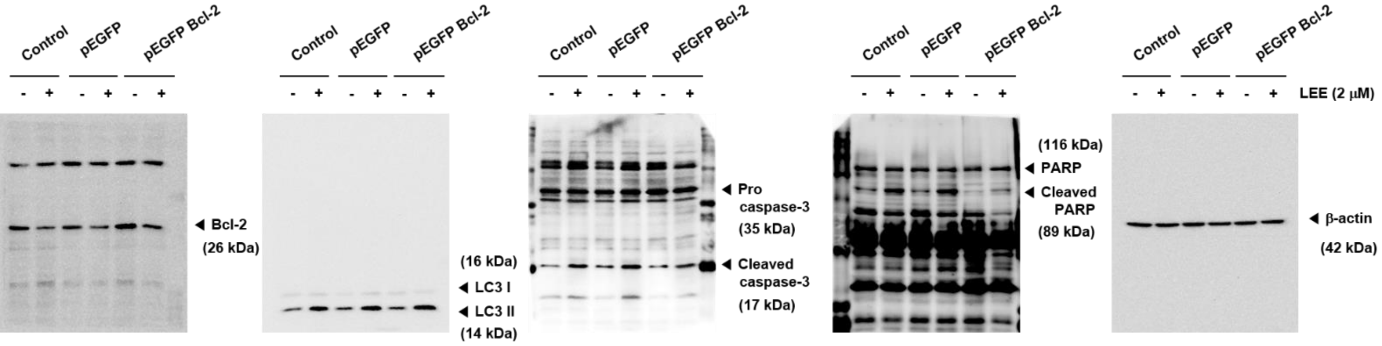

Figure S9. Western blot membrane of Bcl-2, LC3, Caspase-3, PARP, and β-actin proteins were detected with anti- Bcl-2 (sc-492; 1:3000; Santacruz), anti- LC3 (12741s; 1:3000; Cell signaling),anti-Caspase-3 (9661s; 1:3000; Cell signaling), anti-PARP (sc-8007; 1:3000; Santacruz), and anti-β-actin (sc-47778; 1:5000; Santacruz). Proteins were separated on different percentages of gels, and transferred to nitrocellulose membranes (66485; Pall Corporation). Then Membranes were incubated with mouse anti-rabbit IgG HRP (sc2357; 1:5000; Santacruz), mouse-IgGk HRP (sc516102; 1:5000; Santacruz) secondary antibody on room-temperature. Finally membranes were developed with Lumi Femto solution (DG-WF200; 1:1; DoGeNBio)
